# Predicting individual differences in decision-making process from signature movement styles: an illustrative study of leaders

**DOI:** 10.3389/fpsyg.2013.00658

**Published:** 2013-09-24

**Authors:** Brenda L. Connors, Richard Rende, Timothy J. Colton

**Affiliations:** ^1^Chief of Naval Operations Strategic Studies Group, Naval War CollegeNewport, RI, USA; ^2^Department of Psychiatry and Human Behavior, Alpert Medical SchoolProvidence, RI, USA; ^3^Department of Government, Harvard UniversityCambridge, MA, USA

**Keywords:** decision-making, individual differences, hypothetical scenarios, observational methods, movement pattern analysis

## Abstract

There has been a surge of interest in examining the utility of methods for capturing individual differences in decision-making style. We illustrate the potential offered by Movement Pattern Analysis (MPA), an observational methodology that has been used in business and by the US Department of Defense to record body movements that provide predictive insight into individual differences in decision-making motivations and actions. Twelve military officers participated in an intensive 2-h interview that permitted detailed and fine-grained observation and coding of signature movements by trained practitioners using MPA. Three months later, these subjects completed four hypothetical decision-making tasks in which the amount of information sought out before coming to a decision, as well as the time spent on the tasks, were under the partial control of the subject. A composite MPA indicator of how a person allocates decision-making actions and motivations to balance both Assertion (exertion of tangible movement effort on the environment to make something occur) and Perspective (through movements that support shaping in the body to perceive and create a suitable viewpoint for action) was highly correlated with the total number of information draws and total response time—individuals high on Assertion reached for less information and had faster response times than those high on Perspective. Discussion focuses on the utility of using movement-based observational measures to capture individual differences in decision-making style and the implications for application in applied settings geared toward investigations of experienced leaders and world statesmen where individuality rules the day.

## Introduction

Although the idea that individuals can and do differ in how they approach decision-making situations has a long history, recent reviews (Mohammed and Schwall, 2009; Appelt et al., 2011) have called for heightened attention to individual differences in decision-making style. While the majority of published work on decision-making has focused heavily on decision/task features (e.g., complexity, novelty, framing of choice options) and situational/environmental conditions (e.g., time pressure, social context), the importance of paying requisite attention to decision-making style is acknowledged across a number of content areas (Soane and Nicholson, 2008; Mohammed and Schwall, 2009; Weber and Morris, 2010; Appelt et al., 2011). The key notion is that we need to deepen our understanding of lawful variations in the decision-making process across individuals (e.g., Byman and Pollack, 2001; Thunholm, 2004; Del Missier et al., 2010; Harman, 2011; Bruine de Bruin et al., 2012). Or put another way, we need to embrace the idea that *who* is making the decision matters along with the situational and conditional factors that impact how the process of decision-making unfolds.

This argument derives in part from the common sense observation that individuals in the population show considerable variation for most behaviors and traits (Mohammed and Schwall, 2009; Appelt et al., 2011; Bruine de Bruin et al., 2012). With respect to prior research, Mohammed and Schwall (2009) and Appelt et al. (2011) give examples of how individual differences in decision-making style have been observed for phenomena such as omission bias, overconfidence, and simplification strategies. We see promising advances in other areas of research as well, including a deliberate focus on individual differences when studying constructs such as the need for cognition (Carnevale et al., 2010), decision-making competence (Bruine de Bruin et al., 2012), and risk evaluation when making decisions (Harman, 2011; Penolazzi et al., 2013). With these advances, it is evident that an individual differences framework can be a driving force in the decision-making sciences in the same way it has impacted and shaped research in other behavioral domains.

Mohammed and Schwall (2009) offer two suggestions to bolster an individual differences framework when studying decision-making. First, they suggest that a conceptual interest in commonality when observing and recording decision-making behavior be complemented by the careful detection of individual differences across a number of paradigms. They astutely note that there has been a statistical emphasis on means rather than standard deviations in published studies, even though variation around the mean is part and parcel of human behavior (such that the “error” in an ANOVA may in fact be meaningful variance that can be partially explained by systematic individual differences in style or approach). This perspective dovetails with a stream of cogent suggestions—across decades of research—that large individual differences within groups may be obscured when the analytic unit of measurement is the mean (Bowers, 1973; Epstein, 1979; Funder, 2009). Second, they point out that many of the experimental methods used to study decision-making behavior can overwhelm or diminish the impact of individual differences—meaning that other designs need to be entertained. For example, very detailed instructions, strong manipulations within a paradigm, and highly restrictive forced choices (especially two-choice options) can dilute the role of personal characteristics in the experimental setting. It is critical that research methods be employed that can better simulate the real-world context of decision-making, in which individual variation is more clearly prominent (e.g., Bruine de Bruin et al., 2012).

We offer a third consideration. Most attempts to capture decision-making style (e.g., General Decision-Making Style or GDMS; Scott and Bruce, 1995) or cognitive styles (e.g., Rational-Experiential Inventory or REI; Epstein et al., 1996; Norris et al., 1998) have utilized self-report measures. These approaches, and other more recent ones, have yielded constructs such as rational (or slow, deliberate, systematic) vs. intuitive (or fast, superficial) styles—as well as more specific characteristics such as need for closure (Roets et al., 2008) and need for information (Carnevale et al., 2010). In addition, traditional personality measures (such as the Big Five and the Myers–Briggs) continue to be utilized (see Mohammed and Schwall, 2009), but often as a matter of course rather than as measures with clear theoretical ties and proven domain relevance to decision-making style (Appelt et al., 2011). While these measures have content validity and also offer some predictive power (e.g., Carnevale et al., 2010), it can be argued that it would be important to supplement the self-report approach with other methodologies. It is not clear that people are necessarily insightful sources on their own decision-making styles—particularly as the field itself has yet to come to consensus on relevant dimensions that characterize individual styles (Mohammed and Schwall, 2009; Appelt et al., 2011). To this point, many strong arguments have been made that personality science would profit from more overt efforts to measure observable behavior along with self-perceptions (Baumeister et al., 2007; Furr, 2009). Such thinking applies well to the general issue of capturing individual differences in decision-making style.

We propose a complementary approach that is observational in nature and focuses on human bodily movement as a critically important aspect of the decision-making process—and as such offers a unique window into individual differences in decision-making style. The role of the body—and particularly movement—as a fundamental source of cognitive process is receiving increasing attention in scientific circles. The theory of embodied cognition (Wilson, 2002) postulates that cognitive processes are deeply rooted in the human body's interactions with the world and have an inherent connection to movement. A number of recent papers have supported this viewpoint (e.g., Anderson et al., 2012), including some focused on neuroanatomical examinations (e.g., Esopenko et al., 2012). Working from this general theoretical platform, we focus here on one observationally based approach—Movement Pattern Analysis (MPA). MPA is a theoretically based observational methodology that objectively codes body movements of individuals in an interview setting to provide indicators of motivation and decision-making style. MPA is based on the theoretical and applied work of Rudolph Laban, who began study of individual differences in movement and motivation styles in Britain's World War II factories where women had been tapped to replace their men at war and undertake their industrial labor duties. Warren Lamb, his protege further built upon Laban's approach and applied the work known today as MPA to managerial and corporate settings (see Moore, 2005; Lamb, 2012). In Europe and the United States MPA has been used for 50 years with regularity as a tool in the business world for executive recruitment, position selection and the building of management teams, and has been applied in physical and psychological therapies (see Moore, 2005; Lamb, 2012). MPA is also being integrated into the study of world leaders in ongoing work conducted by the Department of Defense (Connors, 2006; Connors, unpublished).

The MPA decision framework assesses processes related to how an individual responds to and acts on the environment. Briefly, MPA centers on the recording of a number of “posture-gesture mergers” (PGMs)—observable behaviors that are considered to correspond to multiple stages of the decision-making process.

A gesture is movement confined to a part (or parts) of the body (some parts of the body relative to movement are isolated from others). A posture is movement that through continuous adjustments of every part of the body becomes consistent as a whole throughout the body. PGMs represent the integration of these two modes, or the flow of posture and gesture into one cohesive quality while the body is moving (Lamb, 2012). PGMs have been shown empirically to correspond with verbal expressions that are authentic, relaxed, truthful, and sincere in contexts that require decision-making (Winter et al., 1989; Moore, 2005; Lamb, 2012)^1^. They have also been replicated by computer experts who model human behaviors to guide the representation of virtual characters (Luo and Neff, 2012).

In the MPA framework, PGMs are used to generate two Overall Factors—Assertion and Perspective—that together represent a signature decision-making style. The core idea is that individuals have a need to balance their actions/motivations devoted to exerting tangible energy in the environment in relation to pressure, time and attention focus to *get results* (Assertion), vs shaping the body (with respect to the cardinal planes of three-dimensional space—horizontal, vertical, and sagittal) to position oneself to *receive from the environment information to create the result* (Perspective). Differences in how individuals achieve their own *balance* between the complementary processes of Assertion and Perspective are proposed to capture different decision-making styles. For example, individuals high on Assertion may employ a mindset of “nothing happens unless I make it happen.” They rely upon decision-making motivations that include intensively focusing to probe and classify information, applying pressure to support determination, and pacing time to implement a decision at just the right moment. In contrast, individuals high on Perspective are more strategic and get results by positioning themselves. They are receptive to a broad scope of ideas and information alternatives—they shape their bodily position to reflect on the decision's relative value or priority and use movements to strategically anticipate the stages of decision implementation to achieve an overall outcome.

These Overall Factors in the MPA framework are thus concerned with capturing individual differences in decision-making style—defined by Appelt et al. (2011) as “… individuals' methods of making decisions” (p. 253) and by Mohammed and Schwall (2009) as “… the unique manner by which individuals perceive, approach, and respond to decision-making situations” (p. 280). The individually-driven balance between Assertion and Perspective in the MPA framework dovetails nicely with potential individual differences in how people in a decision-making setting direct the scope of their information search, size up their situation, develop alternative considerations, and calibrate time pressure to come to a decision. There is reason to believe that individual differences may be most pronounced in precisely these aspects of decision-making (see Mohammed and Schwall, 2009). MPA thus offers a uniquely promising method for observing subjects and deciphering tendencies of theirs that can be used to predict individual differences in future decision-making process.

The overall purpose of this paper is to illustrate a research strategy that explores the utility of MPA as a measure of individual differences in decision-making style that can predict future decision-making behavior. To this end, our outcome measures were derived from tasks that were created to allow for the expression of individual differences in decision-making process. As noted earlier, the predominant paradigms in decision-making science often include design features that diminish the expression of individual differences. We employed hypothetical decision-making scenarios—which have been widely used in both cognitive and political sciences and have shown to have strong linkages to real-world decision-making (Parker and Fischhoff, 2005)—to provide opportunities for subjects to express individual differences. This general idea of recording meaningful individual differences in decision-making process is supported by recent insightful approaches that have shown notable variation in how individuals perform in decision-making situations, such as during the Iowa Gambling Task (Harman, 2011; Penolazzi et al., 2013).

To this end, we permitted subjects the freedom to control their own information search via the option of making requests for more information (for a similar approach, see, for example, Verplanken, 1993), as it is assumed that decision style would be influential in shaping this aspect of the process (Mohammed and Schwall, 2009). Mohammed and Schwall (2009) suggest that decision style should be reflected in the strategies and motivations that guide information search (as some individuals would lean toward acquiring more vs less information before coming to a decision) as well as response time (as those who seek out more information would also spend more time before coming to a decision). These authors refer to information search and response time as features of the “predecisional” stage of decision-making that may be especially reflective of individual differences in decision-making style when assessed using methods that give participants some level of control over these parameters. Of particular note is the following observation:

“Although seemingly commonsensical, these kinds of predictions testing the construct validity of decision styles have yet to be empirically demonstrated in the literature” (p. 298).

In this initial study we examined links between individual differences in the MPA Overall Factors (the balance between Assertion and Perspective) and experimental indicators of decision-making processes (information search and response time) recorded via hypothetical decision-making tasks conducted 3 months after the MPA coding procedure. We recruited a group of seasoned decision-makers—senior military officers with decades of experience (see Mintz et al., 2006). Prior studies have shown notable variation in decision-making style in experienced leaders in general (Carnevale et al., 2010) and military officers in particular (Thunholm, 2004). Since professional background is controlled for when studying experienced leaders, we have an opportunity to illuminate how variation in individual decision-making style plays within a subgroup of individuals who have a history of being faced with similar real-world challenges that rely upon decision-making skills.

Given these considerations, we posed the following questions:

1. Does an inclination toward Perspective (vs. Assertion) predict a greater number of information searches during the hypothetical decision-making scenarios?
2. Does an inclination toward Perspective (vs. Assertion) also predict correspondingly longer response times during the hypothetical scenarios?

## Methods

### Subjects

Twelve current or retired US military officers who had between 20 and 30 years of military service were recruited. All branches of the armed forces (with the exception of the Army), including the Coast Guard, were represented. There were nine males and three females in the group. All subjects provided informed consent in accordance with a protocol approved by the appropriate Institutional Review Board.

### MPA

All subjects participated in a 2-h interview with one MPA interviewer. Following the methodology of MPA, the interview consisted of a series of open-ended questions that focus on life, career history, and present situation. The interviewer records PGMs (as defined in the Introduction) expressed across multiple stages of the decision-making process (see Moore, 2005; Lamb, 2012). The PGMs are initially coded as representing one of six action motivation behaviors that are also representative of one of the two broad dimensions (Overall Factors) of Assertion and Perspective. The proportions of their total PGMs are then tallied across the Assertion and Perspective categories. In this sense the observational system is like a clinical assessment tool in which specific behavioral indicators are recorded and are summed up to generate primary factors. Appendix A describes the MPA action motivations and provides examples of such PGMs that would correspond to Assertion and to Perspective.

Multiple interviewers were used and inter-rater agreement was confirmed for a subset of cases via review by MPA gold-standard coders. Prior research (Winter et al., 1989; Winter, 1992) has shown that PGMs are recorded with high inter-rater reliability. In our study, two MPA raters independently coded the 2-h interview for four of the 12 subjects. As our focus is on the discrimination between the overall factors of Assertion and Perspective, we computed percent agreement for the coders based on this coding decision as applied to the observed PGMs. Across the two coders, 1451 PGMs were recorded and classified as either Assertion or Perspective. Inter-rater agreement was 94.1%. To correct for chance levels of agreement, we computed Cohen's Kappa, which was 0.87, indicating excellent inter-rater agreement (Connors and Rende, in preparation).

Individual differences come into play as individuals find their own balance between the complementary factors of Assertion and Perspective. To this end we created a Perspective/Assertion Balance score—P/A Balance—which we define as follows: % Perspective – % Assertion. This P/A Balance score offers an easily interpretable metric for these data. A score of “0” reflects an individual who allocates equally to Assertion and Perspective; a positive number reflects more distribution to Perspective; and a negative number reflects more distribution to Assertion. Because the data were based on percentages, an arcsine transformation was applied to the data prior to analyses to better approximate a normal distribution.

### Hypothetical decision-making scenarios

Subjects were presented with four hypothetical decision-making tasks (Financial, Health, Voting, and Strategy) in a laboratory setting drawn from a long history of using this paradigm in political science and other behavioral research (e.g., Mintz et al., 2006; Dawes et al., 2007; Gartner, 2008). Subjects were given options to seek out—one at a time—a number of additional pieces of information to consider before coming to a decision. Subjects could either move on to make a final decision, or request another piece of information, in an iterative manner. By way of illustration, Appendix B presents the scenario for the Financial domain and options available for requesting additional information.

During the experiment, the number of information draws—each request for additional information—was recorded electronically for each scenario, as was response time (chronological time measured in seconds). As discussed in the Introduction, the existing literature suggests information search and response time are presumed to be sensitive quantitative indicators of decision-making process that would show differences across individuals. Given our interest in capturing such differences in the process of decision-making—and not the actual decisions being made—we created two summary outcome measures based on subject behavior across all four scenarios. Total Info Draws was computed as the total number of requests for additional information summed across all four hypothetical scenarios. Total Response Time was computed as total chronological time (in seconds) summed across all four hypothetical scenarios.

## Results

Notable individual differences were found for all measures used in this study. Subjects provided a large number of PGMs during the interview along with individual variation. The mean number of PGMs was 155.83 (*SD* = 55.95) − 95.08 coded as Assertion (*SD* = 35.14) and 60.75 coded as Perspective (29.83). The raw P/A Balance score ranged from −60 (reflecting an individual who was coded as 20% Perspective/80% Assertion) to 14 (reflecting an individual who was coded as 57% Perspective/43% Assertion). The total info draws ranged from 10 to 21; total response time ranged from 365.62 to 943.53 s. For each of these measures, there was a relatively equal distribution of scores between the two endpoints noted above.

**Figure 1:**
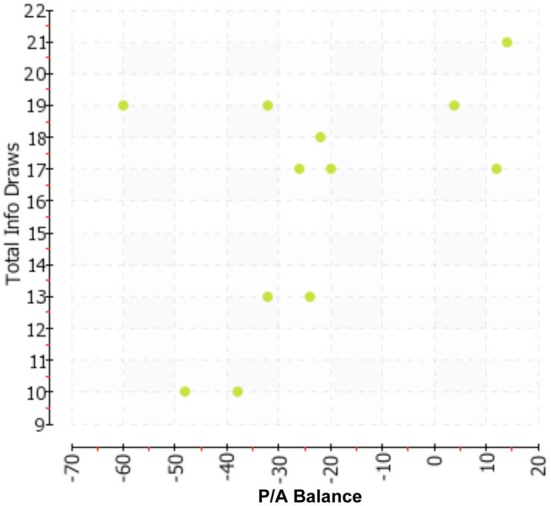
**Scatter plot representing each subject as a data point crossing Total Info Draws (*y*-axis) with P/A Balance (*x*-axis)**.

**Figure 2:**
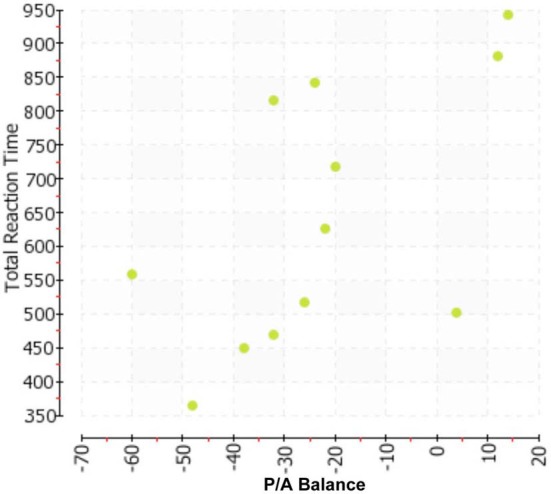
**Scatter plot representing each subject as a data point crossing Total Reaction Time (*y*-axis) with P/A Balance (*x*-axis)**.

To examine the predictive validity of the MPA measure, we computed Pearson correlation coefficients in relation to total info draws, and total response time, respectively. The P/A Balance score correlated 0.50 (*p* < 0.05 one-tail; *p* < 0.10 two-tail) with Total Info Draws, and.61 with Total Response Time (*p* < 0.05 one-tail; *p* < 0.05 two-tail). We present the corresponding scatterplots in Figures 1, 2, which show the strong linear or dose-response association between P/A Balance score and each outcome measure.

The total number of PGMs was not strongly associated with Total Info Draws (*r* = −0.06) and Total Response Time (*r* = −0.15). While somewhat stronger correlations were found between the outcome measures and the total number of Assertion PGMs (−0.30 with Total Info Draws and −0.40 with Total Response Time) and the total number of Perspective PGMs (0.23 with Total Info Draws and 0.18 with Total Response Time), these were not significant given the sample size. Exploratory regression analyses suggested that even after controlling for these raw counts of PGMs, the associations shown in Figures 1, 2 remain significant. For example, the standardized coefficient for the P/A Balance score as a predictor of Total Response Time remained unchanged after adjusting for the PGM counts. We note here that more detailed analyses would be difficult to conduct given the sample size. However, it does appear from this first illustrative study that the P/A Balance score provides substantial traction in the MPA model as a predictor of the experimental decision-making behavior we recorded.

We also note here that the quantitative outcome measures of Total Info Draws and Total Response Time were positively correlated with each other (*r* = 0.54). This is to be expected as a variant of the well-known Hick's law (e.g., Hawkins et al., 2012), which states that the time it takes to make a decision increases as a function of the number of choices that are considered. The individual differences element in this study provides further evidence of the general idea of Hick's law—individuals who sought out more information also tended to have longer response times. As such, we suggest that when individuals are permitted to express individual decision-making styles, lawful relations are observed.

## Discussion

This paper is cast within the current calls in the literature for theory-based investigations of individual differences in decision-making style. Our purpose was to illustrate the potential of using observational methodologies in the service of detecting signature decision-making styles in seasoned leaders that can be used to provide insight into how individuals differ when presented with decisions to be made.

Two related issues deserve mention. First, the observational nature of the MPA paradigm highlights that we do not have to rely entirely on self-report measures to characterize individuals with respect to their “underlying” decision-making style that may predict future decision-making behavior—which again resonates with reminders that observable behavior should be part of measurement strategies in personality science (Baumeister et al., 2007; Funder, 2009; Furr, 2009). The long history of application of MPA in the business world, and more recently in therapeutic settings as well as the political arena of national and world leaders, suggests a real-world validity and utility. Here we illustrate how the MPA methodology can be used in the research setting. We note the intensive nature of the data collection process, as each subject participates in a 2-h interview. It is also important to appreciate the specific expertise of coders who are trained in the MPA and the unique skill and labor-intensive requirement necessary to utilize the data collected in the interview to generate each individual's empirically based profile. Observational work requires rigorous training and extended experience. But we argue that the richness of the data acquired via MPA is well-worth this level of applied focus and energy, particularly when it is both theoretically and practically aimed at capturing decision-making style of, for example, world leaders whose choices can determine whether we have peace or war. While mixing traditionally qualitative research models such as MPA and experimental approaches such as those used in political science pose challenges such an interdisciplinary approach may be the way ahead in terms of bridging worlds of knowledge and testing that they work and reliably predict decision-making. To this end we have illustrated how MPA is capable of measuring variations in how people attend to infomation in effortful vs. reflective ways, and how those characterizations are associated with quantitative tallies derived from experimental protocols.

Second, we also designed tasks to generate experimental outcome data that permitted, rather than muted, the potential for expression in individual differences in decision-making behavior. We suggest that such purposeful methodological approaches will provide a strong platform for future research. We noted earlier the often-used Iowa Gambling Task as an example of a method in which variation in real-time decision-making behavior can be observed and quantified. Similarly, other approaches—such as tasks designed to gauge decision-making competence (e.g., Carnevale et al., 2010; Bruine de Bruin et al., 2012)—demonstrate that there are indeed notable individual differences to be found. Taken together, the message is that altering or creating laboratory-based measures that pull for and measure variation will be of importance in the future.

As this was an illustrative study that required labor-intensive gold standard-trained coders, we presented data on a small—yet informative—sample. That said, our initial data collection effort resulted in convergence between MPA and the real-time quantitative behavioral indicators of decision-making process recorded during performance of hypothetical decision-making tasks. The scatterplots showed a substantial linear association between the MPA constructs of Assertion and Perspective, on the one hand, and well-studied indicators of decision-making style (information search and response time), on the other. These initial findings provide, as recommended by Mohammed and Schwall (2009), the kinds of empirical tests—and associations—that could add much to the growing decision-making literature and support attempts to make more deliberate the study of individual differences. We submit that the robust correlations are due, in part, to the richness offered by methodologies that record observable behavior. Both our predictor variable (P/A Balance) and outcome variables (Total Info Draws, Total Response Time) are real-time phenomena that lend themselves to well-defined recording methods by observers, not participants. While there is often a utility to self-report measures, they cannot be relied on exclusively to record and decipher individual differences. Thus, while the results of this study would need to be replicated with a larger sample, we suggest that these initial findings support that idea that such expanded efforts are worth doing.

Given our emphasis on a methodology that is rooted in observing movement patterns, it is worth expanding on this idea. In general, our approach—and our findings—fit within the theoretical underpinnings of embodied cognition. By capturing PGMs, MPA provides a method for identifying authentic aspects of a person's spontaneous motivation merging the body's postural movement with the movement gestures of the body's parts. As such, it provides a wide-based and dynamic picture of mind-body action and thinking during the decision-making process. For example, highly depressed subjects produce significantly fewer PGMs than control subjects, suggesting that they experience difficulty in being engaged physically and psychologically in ongoing communication (Lamb, 2012).

These ideas of authenticity and motivation are key ones to consider as a part of decision-making style. We postulate that the strong associations reported here reflect the motivational aspects of behavior that are invoked in decision-making contexts. Individuals high on Perspective are more likely to position themselves to explore a broader range of information and in so doing may spend more time to consider a greater breadth of information. Individuals high on Assertion probe information intensively and vary control of their time pacing more overtly to come to a decision and were observed to make their decisions with less information. In the MPA model, Assertion and Perspective are motivational propensities that are balanced differently by different people—there is no valence attached to a score on the P/A Balance spectrum. Rather, MPA offers a method for gaining insight into where on that spectrum an individual resides as a way of understanding how they uniquely balance the complementary roles of Assertion and Perspective in the decision-making process.

MPA provides additional layers of specific qualitative decision-making processes via the complementary action motivation behaviors of each decision stage (see Appendix A). The more granular indicators of motion factors, such as accelerating and decelerating associated with the pacing of time or the preference for linking decision components into longer-term decision staging, could be important predictive measures in studies on negotiating. Other MPA indicators capture decision interaction style during the three stages of decision-making as well as the number of simultaneous novel decision tasks an individual can take on at any one time. Whether a leader is strategic or operational/assertive could become, for example, key measures during terrorist interrogations. The strong associations between the overall factors of MPA—Assertion and Perspective—and laboratory-based measures of decision-making process suggest that future research on MPA may be profitable, particularly in stimulating new approaches to capturing individual differences in decision-making style.

We focused on experienced decision makers (see also Thunholm, 2004; Carnevale et al., 2010) in order to illuminate individual differences in decision-making style. While future approaches could expand this methodology to incorporate individuals of varying experience in decision-making positions, we propose that there may be especially important real-world implications of studying leaders. Developing methodologies that gauge how individuals bring their own intrinsic approach to the decision-making setting would bring new tools to various fields that have vested interest in understanding how leaders make decisions. For example, Mohammed and Schwall (2009) suggest that decision-making is a “… fundamental, ubiquitous process that many fields are actively seeking to improve (e.g., military, medicine, education, government) … ” (p. 250). It has been argued that political science has, to a large degree, ignored the impact of individual personality of world leaders—and how it may transcend contextual factors to be a critical influence on international relations and the shaping of policy (Byman and Pollack, 2001). Application of methods such as MPA may help us better understand with some measurable precision when and how the individual makes a difference in policy making—particularly with respect to how they assert or position themselves with respect to information and responding to time demands in a decision-making crisis. Moreover, MPA measures the tangible physical action pattern as well as motivations. This means more explicitly movement can reveal what leaders may actually do—for example the extent to which they take risk, evaluate priorities and the degree to which cognitive flexibility may underlie a leader's determination. In the world of policymaking insight into an individual's action preference as well as trait and cognitive style is a step forward in understanding the whole picture in today's complex systemic, political, and bureaucratic contexts. To that end, research protocols that examine the predictive validity of methodologies such as MPA have great potential to support application to the real-world issue of detecting the decision-making style of seasoned leaders (see Connors, 2006; Connors, unpublished).

We noted that larger samples and replication would be necessary to establish with more precision the effect size of MPA as a predictor of decision-making behavior. In terms of future directions, it would also be desirable to expand the technique beyond the individual level and into the study of interactive, collective decision-making in a group setting. Analysis could drill down into MPA's six action motivation factors that comprise and more precisely delineate the Overall Factors of Assertion and Perspective and that could both reinforce and refine our understanding in terms of decision-making process prediction herein.

Finally, larger-scale multi-method studies that include both MPA along with self-report measures of decision-making style would further our knowledge by examining convergence and divergence between different methodologies, and perhaps yield insight into the unique predictive power of observational approaches that measure through human movement decision-making motivation and action taking.

### Conflict of interest statement

The authors declare that the research was conducted in the absence of any commercial or financial relationships that could be construed as a potential conflict of interest.

## Footnotes

^1^Lamb (1985) the creator of MPA writes “Observers rating people's behavior for its sincerity and naturalness, have found that the higher the score the greater the PGM in the movement. There is, in fact, a correlation not only between segregation and contrivance, but also between merging and spontaneity, when movement is translated into behavior.” (p. 99).

## Appendix A: examples of assertion and perspective in MPA

Below we describe specific “action motivation” constructs, and present specific examples of behavioral indicators of posture-gesture mergers (PGMs) that are reflective of the primary Overall Factors of Assertion and Perspective. Further details on the MPA model can be found in Moore (2005) and Lamb (2012).

### Assertion—applying effort to make the decision process occur

Assertion involves the tangible exertion of physical and mental effort acting on the environment along three dimensions: direction/focus (associated with giving attention on information search); pressure management (associated with forming intention and priorities by modulating pressure); and time (associated with pacing and staging the implementation of action). The three action motivations reflective of Assertion are as follows:

#### Investigating

Investigating refers to varying focus in space within a prescribed area by probing, scanning, and classifying information. In the interview setting, an example PGM would be zeroing in on the interviewer with head, torso, and a pointing gesture merged.

#### Determining

Determining refers to varying pressure to affirm purpose, build resolve, and forge conviction. In the interview setting, an example PGM would be pressing the body (including seat, hands, and feet) down in a chair followed by and merging into gesturing with the chin.

#### Timing

Timing refers to varying the speed or adjusting the moment-by-moment timing of action, and pacing implementation. In the interview setting, an example PGM would be a very quick gesture that is followed by the whole body shifting rapidly.

### Perspective—shaping to position oneself to create decision process

Perspective has to do with shaping physical perception through changes in the body's convexity or concavity in the horizontal, vertical, and sagittal planes in space. The three action motivations reflective of Perspective are as follows:

#### Exploring

Exploring refers to perceiving the scope available, looking for something new, and being receptive to information from many areas. In the interview setting, an example PGM would be spreading the chest and having that movement flow into the arm gesture in a meandering way.

#### Evaluating

Evaluating refers to weighing up the immediate needs and sizing up the issues. In the interview setting, an example would be to rise up on one side of the body, crossing the legs flowing into the “thinker” pose.

#### Anticipating

Anticipating refers to perceiving the developing stages of action, foreseeing the consequences of each stage and taking some action. In the interview setting, an example would be gesturing while then molding the torso back into a chair.

## Appendix B: experiment decision-making task

We administered hypothetical decision-making scenarios representing four domains: Financial, Health, Voting, and Strategy. Each scenario used a similar format: a decision-making task is given to the subject along with the option to seek out specific types of additional information. These can be selected in any order and the subject can move on to make a decision at any time and consult however many additional pieces of information they want (including none). Other options are provided once an initial decision is registered. Below one example (Financial) of the hypothetical scenarios used in this study is presented.

### Financial

Imagine you just inherited a million dollars from an uncle who died after a long and trying bout with diabetes and kidney failure. You were close to your uncle and took very good care of him during his final illness, visiting often, and helping with medical decisions, but you did not handle his finances and were quite surprised after the will was read to discover he was so well-off. You are now confronted with how best to invest this money. Because of the nature of the trust left to you, there are certain restrictions on how the money can be invested. Due to arcane aspects of the tax law, you can only invest in certain stock mutual funds and particular bond funds, and you can only buy real estate worth more than $200,000. You now need to decide what to do with the $1,000,000 bequest.

Additional Information There is additional information available. Please select as many additional categories of additional information below that you would like to read about.

- Information about your own financial situation (1)
- Information about expert projections (2)
- Information about past performance (3)
- I am comfortable deciding now (4)

#### If “information about your own financial situation” is selected:

It may be helpful to keep in mind that you work extremely hard and earn $100,000 a year. However, you have a lot of financial demands made of you, and so you have not been able to amass any savings. You do not currently own any stocks or bonds and you rent your nice but modest apartment. Your fixed expenses are $84,000 a year, with much of that going to taxes, your parents' rent, and your children's college tuition.

#### If “information about expert projections” is selected:

Here are some forward projections relevant to your investment options, provided by financial experts.

***Real estate:*** Prices have recently slumped in the real estate market. Experts believe they will take a minimum of 10 years to fully recover to their pre-slump level, and are likely to increase modestly after then. If you purchase a house and sell it before that time, there is a good chance you will lose part of your equity. However, you will benefit from the tax deductions of the mortgage interest in the meantime. You may also benefit from the value of living in any house you buy during this time.

***Stocks:*** This is a high-risk option which can offer potentially high payoff. Although recent declines in the market have lowered overall value, the long-term prospects for capital growth over the coming decade appear to be about 10% a year. However, if political unrest, economic catastrophe, or environmental disaster occurs, you could easily lose your entire investment.

***Bonds:*** This is a low-risk, low-return option. While you are very unlikely to lose your initial investment, your long-term growth over the coming decade will not likely be more than 2 or 3% a year. In addition, you will not have easy access to your capital before the bonds come due in 10 years and will pay a heavy penalty if you try to turn in the bonds before then.

***Money in the bank:*** This would give you the greatest security and liquidity of the four options. If your money is put into separate deposits up to the allowable limit of $250,000, it will be fully insured by the federal government. If the capital is deposited this way, it will also be easy to access it at a moment's notice. However, your rate of return can be expected to be extremely low, less than 1% per year.

#### If “information about past performance” is selected:

Here are some facts about past performance of your several options.

***Real estate:*** Over the last 10 years, the value of property in your area has increased 500%. However, in the last year, such values have declined by a factor of about 25%.

***Stocks:*** Over the last 10 years, the value of the mutual fund that is available to you has increased by about 100%. However, in the last year, it has decreased by 20%.

***Bonds:*** None of the bond options available to you has defaulted in the past. Overall interest rates for the bonds you can buy with this money have been 2–3%. The penalty rate for turning in the bonds before the maturity date has historically been 50%.

***Money in the bank:*** The average annual interest rate over the last 10 years has been 0.8%.

#### If “i am comfortable deciding now” is selected:

Of your $1,000,000 inheritance, how much would you like to allocate to the following categories? Your allocation should sum to $1,000,000.

**outcome1_1** Residential real estate (minimum investment: $200,000) ______

**outcome1_2** Stocks ______

**outcome1_3** Bonds ______

**outcome1_4** Keep cash in the bank ______

You have now indicated how you would prefer to distribute your inheritance. Let us suppose, however, that several items of breaking news have become available, which may be relevant to your investment choice. Would you like to learn what those items are, or would you prefer to stick with your original choice?

- Learn about breaking news (1)
- Stick with choice (2)

#### If “learn about breaking news” is selected:

***Breaking News:*** In the last few weeks, the euro, the currency of the European Union, has been shaken by a crisis of confidence owing to Greece's default on its national debt and unexpected economic turbulence in Germany and France. There is discussion in the media of possible spillover effects into the United States and the global economy. The Federal Reserve Board is going to meet next week to reconsider the US discount rate. In light of this news, would you like to reallocate your money?

- Yes (1)
- No (2)
